# Growth on Hydrogen by the Sulfate-Reducing *Oleidesulfovibrio alaskensis* Induces Biofilm Dispersion and Detachment—Implications for Underground Hydrogen Storage

**DOI:** 10.1021/acs.est.4c13893

**Published:** 2025-04-04

**Authors:** Na Liu, Christian Ostertag-Henning, Martin A. Fernø, Nicole Dopffel

**Affiliations:** † Department of Physics and Technology, University of Bergen, Allegaten 55, 5007 Bergen, Norway; ‡ Bundesanstalt für Geowissenschaften und Rohstoffe, Geozentrum Hannover Stilleweg 2, 30655 Hannover, Germany; § Norwegian Research Centre AS—NORCE, Nygårdsgaten 112, 5008 Bergen, Norway

**Keywords:** sulfate-reducing bacteria, bioclogging, microbial dynamics, underground hydrogen storage, biofilm detachment

## Abstract

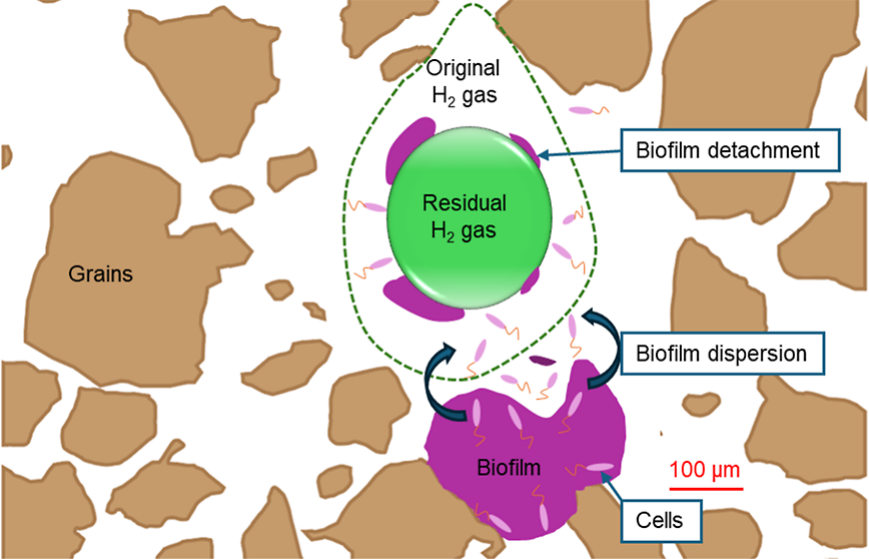

Hydrogen is a versatile energy carrier for human activity but is also a ubiquitous electron donor for subsurface microorganisms. During underground hydrogen storage operations, it is expected that microbial communities will use the injected hydrogen as electron donor for diverse metabolisms, and induce a variety of microbial-triggered risks. A significant concern is the formation of biofilm and induced bioclogging, which may reduce the hydrogen injectivity and storage operation efficiency by altering the subsurface hydrogen flow. This study investigates how different electron donors—specifically hydrogen and lactate—affect the growth dynamics of a sulfate-reducing bacterium (*Oleidesulfovibrio alaskensis* G20) and the associated biofilm formation in porous media. The pore-scale observations reveal that lactate promotes robust biofilms resulting in bioclogging, compared to hydrogen promoting increased microbial motility with less biomass production. Potential hydrogen chemotaxis leads to biofilm dispersal and detachment over time as the cells seemingly favor a planktonic lifestyle over biofilm formation. Multiple hydrogen injections enhanced biofilm detachment and reduced the risk of pore blockage associated with microbial growth. Three hydrogen injections resulted in 69% biofilm detachment, while nitrogen injection caused only 31% detachment over three cycles. The combination of increased cell motility and reduced biofilm attachment indicates that the risk of bioclogging during cyclic UHS operation might be low for this model bacterial strain.

## Abstract 2

Hydrogen promotes biofilm dispersion and detachment in porous media when used as an electron donor for *Oleidesulfovibrio alaskensis*. This finding is significant for underground hydrogen storage, as it alleviates the expected risks of biofilm formation and pore blockage, enhancing the technology’s efficacy in the clean energy transition.

## 1 Introduction

To bridge the gap between daily fluctuating renewable energy production and times of energy demand, research into effective energy storage methods is critical.^1^ Green hydrogen (H_2_) produced via electrolysis of renewable electricity can be used as an energy carrier and be stored for longer durations to balance energy generation and use. H_2_ has promising characteristics like its high mass energy density (33.3 kW h/kg), but its low volumetric calorific value (3 kW h/m^3^) necessitates large-scale storage options.^2^ Underground H_2_ storage (UHS) in subsurface reservoirs like saline aquifers and depleted hydrocarbon reservoirs offers an economic solution. Studies and projects^3,4^ have tested and established the technical feasibility of storing H_2_ in these formations. One important aspect for successful UHS is understanding the potential microbial consumption in the subsurface.^5,6^ H_2_ gas is a versatile electron donor for many subsurface microorganisms, and adverse microbial activity can cause H_2_ gas loss, operational risks due to H_2_S formation and detrimental changes to reservoir properties.^6−8^ A significant concern is the formation of biofilms on mineral surfaces in the pores of the reservoir rock, which can lead to bioclogging.^5^ Bioclogging or bioplugging is particularly complex in UHS, as it may block H_2_ gas upward flow, leading to more uniform radial gas penetration into the reservoir—potentially benefiting gas storage security.^9^ However, the presence of clogged pores can also cause substantial damage to the reservoir and lead to decreased gas injectivity and recovery, which may negatively impact the storage. While previous studies have explored biofilm formation in porous media, the specific mechanisms of biofilm development, motility-driven dispersion, and detachment under cyclic injection/production conditions in UHS remain poorly understood. In particular, the role of different electron donors in influencing microbial adhesion, biofilm resilience, and dispersion dynamics has yet to be fully elucidated. Therefore, improved understanding of microbial growth dynamics and biofilm formation with H_2_ gas is essential for optimizing reservoir performance and effectively mitigating microbial risks that could impact the safety, efficiency, and longevity of UHS systems.

During UHS operations, three primary risk groups of microorganisms can use the stored H_2_ gas as an electron donor: hydrogenotrophic sulfate-reducing bacteria (SRB), homoacetogenic bacteria, and hydrogenotrophic methanogenic archaea.^5^ Especially SRBs are of major concern as they are known to form dense biofilms and their end-product is the toxic and corrosive H_2_S gas.^10^ The presence of H_2_S can cause economic and environmental issues, including reservoir souring, infrastructure degradation, and increased operational risks during gas storage and its migration is also hazardous to human health.^11^

SRBs are prevalent in subsurface environments and can utilize H_2_ gas in the following reaction^12^
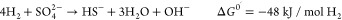
1

The standard Gibbs free energy yield (Δ*G*^0′^) marks the thermodynamic favorability of a reaction at ambient pressure and temperature, pH 7 and 1 M of all reactants.^7^ The OH^–^ production leads to an increasing alkalinity in the surrounding environment, a side-effect that is specific for H_2_-oxidation and will not occur when SRBs grow in the presence of other electron donors, such as lactate. Recent salt caverns studies have demonstrated that SRBs consume H_2_, leading to an increase in pH and a gradual reduction in microbial activity over time.^13^ Self-limiting microbial activity as a result of increased alkalinity also raises questions about the long-term impact from biofilm formation on the UHS efficacy.

Lactate, a common electron donor for SRBs in subsurface environments with abundant organic carbon, has been extensively studied as an organic substrate for enriching SRB populations in laboratory settings.^14^ Many SRB species utilize lactate not only as an electron donor but also as a carbon source during sulfate reduction. This dual role enhances their metabolic efficiency and contributes to their proliferation in specific environments leading to often dense biofilm formation.^12^ The metabolic process can be represented by the following reaction
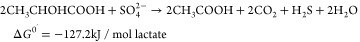
2

The production of acetate (CH_3_COOH), CO_2_, and H_2_S during these metabolic processes can lead to a decrease in the pH of the surrounding environment, in contrast to H_2_ oxidation. As a result, different electron donors can lead to significantly varying yields of bacterial biomass and growth behavior. For example, studies conducted in laboratory-scale gas-lift reactors have demonstrated that using H_2_ as an electron donor results in significantly lower biomass production compared to using lactate.^12,15^ Considering that during UHS, H_2_ will be the main electron donor, it is unclear how the growth behavior of SRBs influences the reservoir properties and if biofilm formation will actually occur. In this study, we employed a model SRB *Oleidesulfovibrio alaskensis* (formerly *Desulfovibrio alaskensis*)^16^ to examine the differences in microbial activity, growth and biofilm formation when exposed to H_2_ or lactate. This investigation aims to clarify how these electron donors influence planktonic and sessile (biofilm) lifestyles and how this relates to potential microbial-induced pore clogging during UHS.

## 2 Materials and Methods

### 2.1 Microorganisms and Culture Conditions

The Gram-negative, sulfate-reducing model bacterium DSM 17464 *O. alaskensis* G20 was used in this study. It exhibits a growth range spanning pH 6.5 to 8.5, temperatures between 10 and 45 °C, and can thrive in NaCl concentrations ranging from 0 to 10% (w/v). *O. alaskensis* reaches its peak growth rate under optimal growth conditions in marine Postgate medium at 37 °C, with a pH of 7.0 and NaCl 2.5% (w/v) under anaerobic conditions.^17^ It can use lactate or H_2_ as an electron donor and sulfate as an electron acceptor to produce H_2_S for growth. Acetate was used as carbon source when growing on H_2_. For cultivation purposes, a modified DSMZ 195c growth medium was used with reduced salt content as a base medium, consisting of: 0.02 M Na_2_SO_4_, 0.005 M NH_4_Cl, 0.001 M KH_2_PO_4_, 0.36 M NaCl, 0.01 M MgCl_2_·6H_2_O, 0.007 M KCl, 0.001 M CaCl_2_·2H_2_O, 1.0 g/L trace element SL-10, 0.25 mL/L Na-resazurin solution (0.2% w/v), 0.01 M Na_2_CO_3_, 0.002 M Na_2_S·9H_2_O and 10 mL/L vitamin solution. The pH of the medium was adjusted to a range of 7.1–7.4 using Na_2_CO_3_ and HCl. The bacterial suspension was inoculated by adding 10% (2.5 mL) of a concentrated bacterial solution (grown on 0.021 M Na-lactate) into 25 mL of the base medium. To further support bacterial growth, an additional 0.021 M Na-lactate and 0.02 M Na-acetate were added to the bacterial suspension. The 50 mL bottle, containing 27.5 mL of liquid and 22.5 mL of headspace, was incubated at 37 °C for 3 days under anaerobic conditions, with a N_2_/CO_2_ mixture (80:20) in the headspace. The bacterial suspension was then used as an inoculum for biofilm growth and microfluidic experiments with addition of different electron donors.

### 2.2 Biofilm Growth in μ-Dish and μ-Channel Experiments

The bacteria were incubated under anaerobic conditions using an anaerobic chamber with Na-resazurin added as an oxygen level indicator—when the solution turns pink, the oxygen concentration exceeds the acceptable threshold and the solution was not used further. Both the μ-dish (ibidi GmbH) and the μ-channel (μ-channel Luer channel slides, ibidi GmbH) systems were operated at atmospheric pressure, and the maximum H_2_ concentration in the solution was calculated based on H_2_ solubility under experimental conditions (∼0.78 μM).^18,19^ Transferring the bacterial solution (buffered with Na_2_CO_3_) to the μ-dish/μ-channel setups in the absence of CO_2_ will result in a slight pH shift until a new equilibrium was established. The airtight systems, combined with the small gas volume (<2 mL), kept the CO_2_ outgassing to a minimum. Consequently, any pH variations resulting from gas exchange were negligible compared to those driven by microbial activity.

#### 2.2.1 Microbial Growth in the μ-Dish

After incubation, the μ-dish (surface area 3.5 cm^2^) was transferred into a stage top incubator (H101–K–FRAME, Okolab) while maintaining anaerobic conditions with a continuous flow of N_2_ gas and a constant temperature at 37 °C using a temperature controller (H101-CRYO-BL-T, Okolab). Real-time imaging was performed using a Nikon Eclipse Ti2 microscope in DIC phase contrast using a high-speed ORCA Fusion camera (Figure S1). Biofilm formation was studied for the two different electron donors and a control: (i) lactate (0.021 M lactate, 0.02 M acetate), (ii) H_2_ gas (pure H_2_ + 0.02 M acetate), and (iii) No donors. Note that the lactate concentration (0.021 M, significantly higher than that in real storage sites) was used to ensure measurable microbial activity and to allow for a clear comparison with hydrogen-based metabolism.

#### 2.2.2 Microbial Cells Sensitivity in the μ-Channel

Microbial cells sensitivity to H_2_ gas was assessed using an anaerobic μ-channel with a length of 50 mm, a width of 5 mm and a depth of 0.4 mm (Figure S2a). After initial incubation, pure H_2_ gas was injected into the channel at ambient pressure using a gastight syringe. One end of the channel was filled with the bacterial solution, while the other end was filled with pure H_2_ gas, creating a distinct gas/liquid interface in the middle of the channel. The microchannel was incubated in the heating cabinet at 37 °C for approximately 18 h to allow for initial growth and acclimation. Following the initial incubation, the channel slide was transferred to the stage top incubator set to the optimal culturing temperature, ensuring consistent environmental conditions for microscopic imaging analysis. The microbial activity and the number of moving cells along the gas/liquid interface were assessed by capturing videos at isolated locations to monitor changes in cell behavior and viability in response to H_2_ gas exposure.

### 2.3 Biofilm Dynamics in Porous Media

Porous media microbial growth dynamics and biofilm formation with different electron donors were studied experimentally. Pore-scale observations were enabled using a high-pressure microfluidic pore network etched on a silicon wafer with a borosilicate glass top. The porous media was 36 repetitions of a unique pore pattern from a natural sandstone, with a total porosity of 0.61 and pore volume of 11.1 μL (etching depth: 0.03 mm; length: 27 mm; width: 22 mm).^20^ Pore-scale displacement event and microbial activity were imaged with a Zeiss microscope (Axio Zoom. V16, Zeiss) illuminating with an S ring cold-light (CL 9000 LED) source. The system temperature kept constant at 37 ± 0.5 °C by circulating water through internal copper tubes in the chip holder. Experimental pressure was controlled at 10.55 barg with a high precision plunger pump (Quizix Q5000–10 K) and a back pressure regulator (EB1ZF1 Equilibar Zero Flow) connected to a pressurized 300 mL N_2_ cylinder. An in-depth description of the experimental setup and porous media microbial inoculation can be found in the Supporting Information (Figure S3).^21^

#### 2.3.1 Biofilm Formation with Lactate and H_2_ Gas

Porous media biofilm formation was studied using the same electron donors (lactate and H_2_). For lactate-induced biofilm growth, a mixture of bacterial solution and media with added 0.021 M lactate solution in a 1:1 volumetric ratio was inoculated. A continuous feed of lactate solution at 1 μL/min was used to promote biofilm growth within the pore network, delivering 0.021 M of lactate per minute with an average flow velocity of 0.09 mm/min. For H_2_-induced biofilm growth, the bacterial solution was incubated in the pore network for 16 h to allow initial attachment, after which H_2_ was injected at 5 μL/min until gas breakthrough was observed at the outlet. Subsequently, 100 pore volumes (PVs) of H_2_ were injected at the same rate, with the pore pressure maintained at 10.55 barg.

#### 2.3.2 Biofilm Detachment during Multiple Gas Injection

Biofilm detachment was evaluated through a multistep H_2_ injection experiment. The mixture (1:1 bacterial solution + lactate solution) was inoculated into the pore network for 3 days for initial biofilm formation. Pure H_2_ was then injected at 5 μL/min until the gas breakthrough occurred. Following the breakthrough, an additional 100 PVs of H_2_ gas were injected to ensure the concentration of dissolved H_2_ in the aqueous phase stabilized at the experimental temperature and pressure (cf. Supporting Information S1).^22^ Once the equilibrium was reached, the outlet was closed to maintain constant pore pressure (10.55 barg) for 7 days, permitting H_2_ consumption and biofilm growth. Two additional H_2_ injections were performed at 7 day intervals to assess biofilm detachment over time, following the same procedure. The H_2_-induced biofilm growth was benchmarked against N_2_, following an identical multistep experimental procedure. Due to the small volume of our microfluidic pore network (11 μL), we were unable to extract sufficient effluent for further analysis. All H_2_ injection experiments were repeated three times to assess reproducibility.

### 2.4 Image Acquisition and Analysis

Image acquisition for the μ-dish system used a time-programmed sequence with the following three data points acquired every 60 min: one overview 2D XY image (10× objective); a stacked slice deck (60× objective); 5 s video to identify motile bacterial cells. To assess three-dimensional bacterial growth dynamics, the slice deck consisted of 43 2D XY images with 0.11 μm pixel distance between 0 and 25.2 μm above bottom surface of the μ-dish (depth difference of 0.6 μm). For the μ-channel system, 5 s videos at the frame rate of 20 frames per second were recorded at five different positions along the gas/liquid interface on day 1 and 4. Image acquisition for the microfluidic system used an image-stitching approach to capture the whole pore network (total 121 separate images) with a resolution of 1.1 μm/pixel and acquisition time of 73 s.

**Figure 1:**
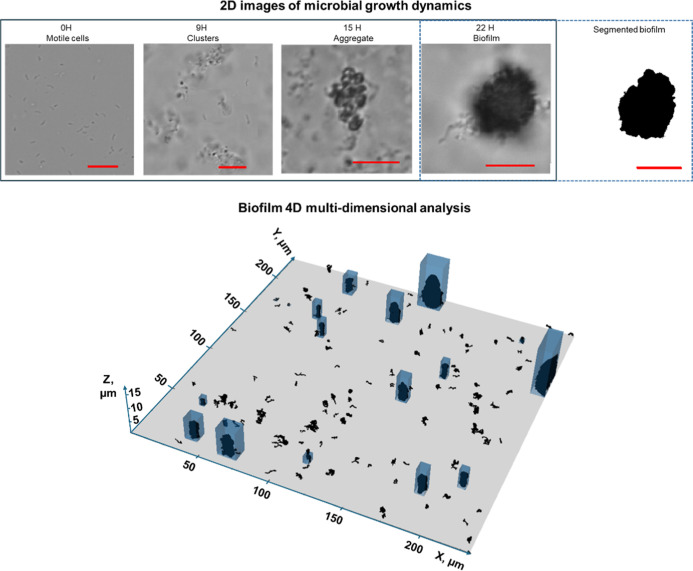
Image acquisition to analyze cell growth and biofilm formation. Time series (top row images) demonstrating the development (left to right) from individual motile cells (0 h) into clusters (9 h) and aggregates (15 h) to form well-structured communities referred to as biofilms (22 h). The segmented binary image (rightmost image) was used to quantify 2D spatial distribution. The three-dimensional biofilm structure (bottom image) was acquired over time to quantify temporal biofilm growth, surface attachment and spatial distribution by stacking segmented binary 2D images along the *Z*-axis. Blue boxes are overlain larger biofilms structures as illustration. Red scale bar is 10 μm for all 2D images.

Image segmentation was employed for all systems to obtain quantitative data on the size and distribution of microbial cells and biofilms. The image analysis method used for the microfluidic system is described in the Supporting Information (Figure S4). Figure 1 illustrates the image processing steps for images from the μ-dish systems. Grayscale microscope images were converted to binary images via global thresholding, employing the Multi-Otsu algorithm from scikit-image library.^23^ The segmented 2D images were then stacked sequentially along the *Z*-axis to reconstruct the 3D biofilm structure. Distinct features within each slice were identified and labeled, and the feature areas were extracted using the label and regionprops functions from the skimage.measure module. This enabled filtering to exclude single cells or irrelevant structures, ensuring that only biofilm-relevant regions were retained. Subsequently, the refined 3D volume was processed using the marching cubes algorithm from scikit-image library, which generated a surface mesh to capture the biofilm’s intricate 3D structure. This mesh, consisting of vertices and faces, was visualized using the Poly3DCollection in matplotlib, enabling detailed visualization and structural analysis of the biofilm. The spatial position of each biofilm was tracked over time by recording its coordinates, allowing for temporal analysis of biofilm development within the solution.

The image segmentation of moving cells in the solution was performed using the Trackpy library for particle tracking and analysis. The recorded video frames were processed to identify cells as particles based on contrast with the background. Trackpy’s locate function detected the cells’ positions, and the track function linked them across frames, enabling tracking of individual cell movement. The velocity field of the moving cells was computed by analyzing the displacement of tracked cells over time, providing insights into flow dynamics and microbial motility within the system.

## 3 Results and Discussion

### 3.1 Microbial Growth Dynamics with Different Electron Donors

Microbial growth kinetics are highly dependent on substrate availability, particularly the presence of electron donors. In this study, we investigated microbial growth dynamics using in situ microscopic analysis in a μ-dish (Figure S1) and a μ-channel setup (Figure S2a). This method enabled real-time observation of microbial cell proliferation, the transition from planktonic to sessile states, and subsequent biofilm formation. Our findings revealed distinct growth patterns depending on the type of available electron donor.

#### 3.1.1 Microbial Cells Response

**Figure 2:**
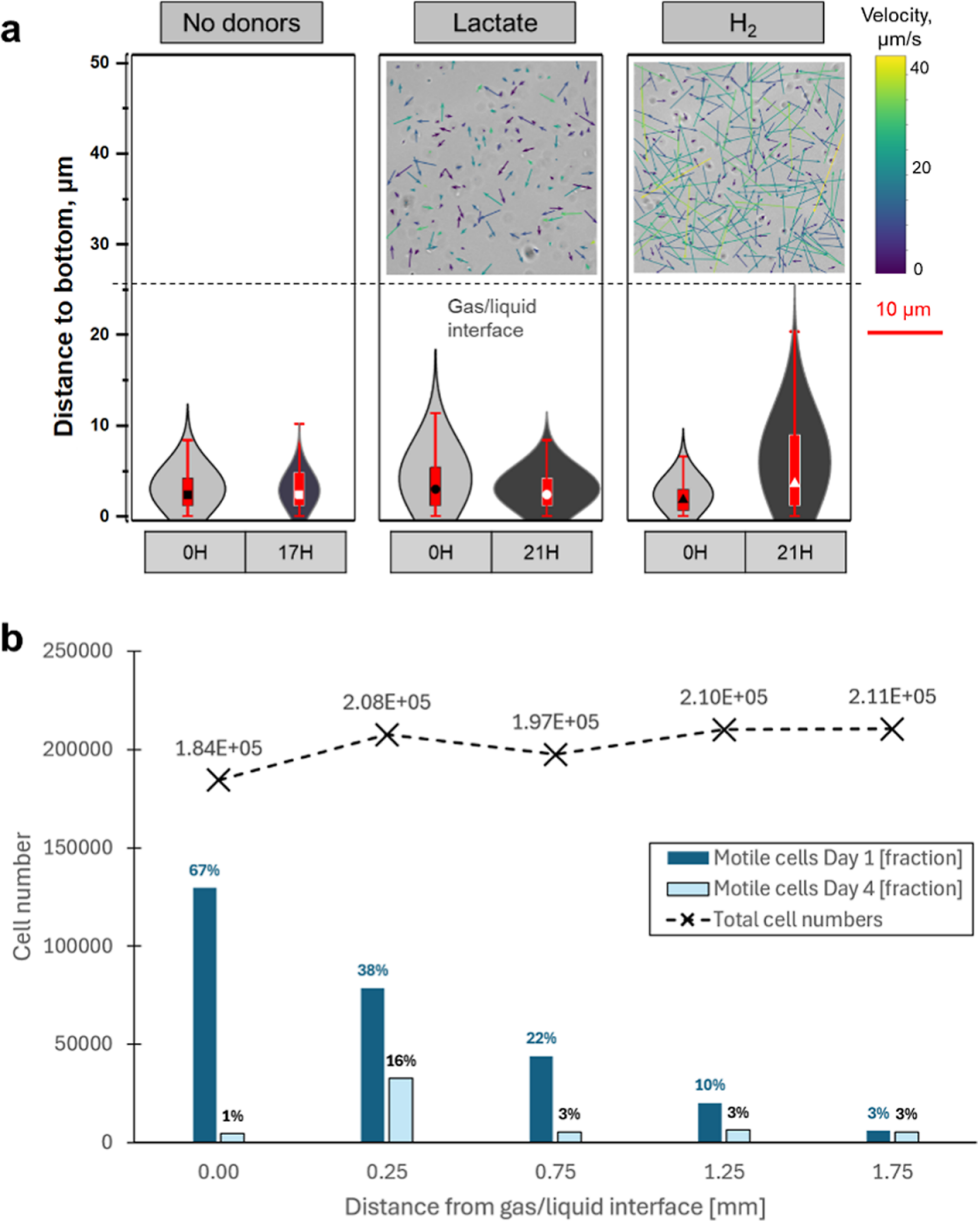
Microbial cell distribution, response and metabolism for different electron donors. (a) Vertical microbial cell distribution for three electron donor cases. The central red boxes within each violin represent the interquartile range (encompassing 25% to 75% of the cells), while the symbol within each box indicates the median value of cell distribution. Insert images: The velocity field plots compare microbial cell mobility in lactate and H_2_ conditions. (b) Cell motility as a function of distance from H_2_ electron donor. Total cell numbers were recorded on day 1, with most cells having settled at the bottom of the μ-channel on day 4. The number and fraction of motile cells decreased (from 67% to 3%) with distance (0–1.75 mm) from the H_2_ electron donor at the gas/liquid interface. The fraction of motile cells generally decreased (from an average of 28% to 5%) over time (4 days), with one notable exception: the presence of biofilms (from the preculturing solution) in the region adjacent to the g/l interface (0.25 mm distance) preserved motile cells better compared with all other regions. In the most remote region (1.75 mm from g/l interface) the fraction of motile cells was low and consistent over time (2.6% to 2.8%).

The distribution and behavior of microbial cells in the solution varied significantly based on the available electron donor across the three experiments (Figure 2a). Without electron donors, cells remained largely stationary at the bottom. With lactate as their electron donor, cells settled near the bottom (<10 μm) due to nutrient abundance. In contrast, cells migrated toward the gas/liquid interface (located 25.2 μm above the bottom surface) to access H_2_ as their electron donor. Beyond the differences in cell distribution between the lactate and H_2_, significant differences in cell motility were observed. With lactate, cells showed limited movement (max velocity: 9.5 μm/s, average: 3.7 μm/s, SD: 2.3 μm/s). Cells in the H_2_ environment had a higher average velocity (14.9 μm/s) and maximum velocity (43.7 μm/s) with a standard deviation of 9.9 μm/s, indicating increased motility and faster movement in the presence of H_2_. Higher motility is likely associated with substrate availability: lactate, being uniformly dissolved in the solution, is readily accessible to cells; whereas the low H_2_ solubility in water (less than 0.72 mM) requires cells to actively migrate toward the gas–liquid interface where H_2_ is replenished. Flagellar motility is a critical ability for nutrient acquisition, biofilm formation and escape from stress (e.g., temperature, pH, malnutrition) for many different microbes.^24^ The motility of *O. alaskensis* is described to be motile to be driven via a single polar flagellum.^17^ We suspect that flagellar biosynthesis is likely upregulated in combination with chemotaxis pathways during H_2_ oxidation and subsequent H_2_ limitation. This will enable cells to seek areas regions with higher H_2_ concentrations to sustain active sulfate reduction.^25^ Further tests are needed incorporating methods on cellular level to directly assess motility gene expression or protein synthesis via qPCR, proteomics or transcriptomics, to elucidate the cellular response to H_2_.

Using the μ-channel (cf. Figure S2a for experimental setup) it was possible to quantify that cell motility decreased with distance from the gas–liquid interface (see Figure 2b). Over time (4 days) the fraction of motile cells decreased, particularly at the interface, as cells settled at the bottom due to adverse conditions (predominantly the pH increase).^13,22^ The final pH in the μ-channel system, though not quantified here, can be inferred from bottle tests with the same strain.^13^ In these tests, the pH can reach values greater than 9 when growing on H_2_ over similar time scales,^13^ consequently exceeding the pH limit reported for this microbe.^17^ This could, therefore, explain the reduced activity. A few biofilms were detected close to the gas–liquid interface (within a 0.25 mm distance). These biofilms, originating from the lactate-grown preculture, were visible right from the start of the experiment (see Figure S2a). The protective advantage of biofilms against environmental stressors^26^ was observed with the highest motile cell fraction (16%) after 4 days (see Figure 2b), suggesting that biofilm formation maintains cellular activity even under challenging elevated pH levels. Throughout the experimental period with H_2_ gas, no new biofilms developed. During the experiment, H_2_ and lactate concentrations in the solution were not measured. Decrease in the sulfate concentration, however, provided evidence for active sulfate facilitated by H_2_ as the electron donor, as confirmed by Raman spectroscopy (Figure S2b).

#### 3.1.2 Microbial Preferred Living Styles

**Figure 3:**
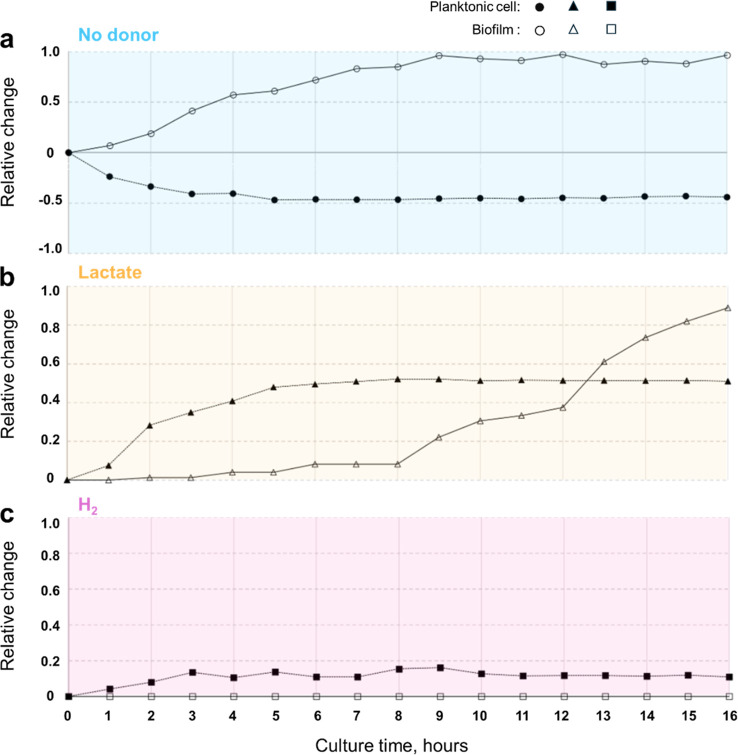
Quantitative analysis of preferred microbial lifestyles with different electron donors. The relative change in biofilm quantity and cell count was calculated by dividing the value at a given time point by the initial value at the start of the experiment. Without electron donors (a), biofilm increased from the onset as planktonic cells merged into biofilm structures. In the lactate experiment (b), planktonic cells proliferated rapidly during the first 6 h, achieving a growth rate of 83.44%. Biofilm formation only began once planktonic cell growth slowed. By 8 h, biofilm growth increased significantly, though a slight decline was later observed, likely due to the merging of smaller biofilm clusters into larger structures. In the H_2_ experiment (c), planktonic cell numbers increased modestly within the first 4 h, with a slower growth rate of 14.15%. No new biofilm formation or growth was observed under the H_2_ condition.

As described above, microbial populations exhibit distinct growth preferences as either planktonic cells or biofilms, largely determined by the availability of carbon sources, electron donors and environmental stress factors.^26,27^ Biofilm formation is a reactive response to environmental changes, where bacterial transition from planktonic growth to surface adhesion under suboptimal or fluctuating conditions, forming structured communities as a survival mechanism.^28,29^ This shift is often triggered by factors such as nutrient depletion, oxidative stress, or the accumulation of metabolic waste. Without an electron donor (Figure 3a), planktonic cell numbers decreased, while biofilm formation increased, indicating that biofilms provide a survival advantage under starvation and oxidative stress. In contrast, the lactate (Figure 3b) and H_2_ (Figure 3c) experiments revealed a preference for initial planktonic growth as cells began consuming the electron donors to form biomass. The lactate triggered significantly higher cell proliferation compared to H_2_, likely due to lactate providing more electrons per mmol and additionally serving as a carbon source. After 8 h, biofilm formation with lactate rapidly increased, indicating nutrient depletion or other growth limitations. With H_2_ as sole electron donor biofilm formation is notably suppressed throughout the experiment. No new biofilm formation was observed. Existing biofilm clusters, introduced via the inoculation from the preculture, also did not show further growth. This supports the findings of our μ-dish experiments with increased and prolonged single cell motility within a H_2_-concentration gradient. The missing biofilm development with H_2_ gas at experimental conditions is likely linked to the combined effect of increased motility and increased pH during microbial H_2_ oxidation. Alkaline pH is known to interfere with initial bacterial adhesion and subsequent biofilm formation for other microbes.^30^ An increase in pH can alter the surface charge of bacterial cells, reducing their ability to adhere to surfaces and affecting the electrostatic interactions crucial for biofilm attachment.^31^ While it could be argued that an increasing pH would trigger biofilm formation as a protective mechanism, our experimental system shows this is not the case when H_2_ is used as an electron donor. Hence, the potential chemotaxis caused by H_2_ sensing seems to overwrite the biofilm triggers, as evidenced by the increase cell motility near gas/liquid interface (Figure 2). Further analysis specifically looking at cellular- and genetic levels are needed to improve our understanding of chemotactic behavior toward H_2_.^32^ Although outside the scope of this study, testing several motility mutants of this strain, combined with gene expression studies, would shed light on the underlying genetic effects.

### 3.2 Biofilm Growth in Porous Media

Biofilm formation in subsurface porous media, like aquifers or oil and gas reservoirs, can lead to so-called bioclogging.^33^ The biofilm accumulation impacts multiphase flow through the network of connected pores by changing the effective pore size and permeability. The effects of planktonic growth versus biofilm under H_2_ flow in porous media were studied using the cultured *O. alaskensis* strain with different electron donors in a reservoir-realistic microfluidic pore network.

#### 3.2.1 Bioclogging with Different Electron Donors

**Figure 4:**
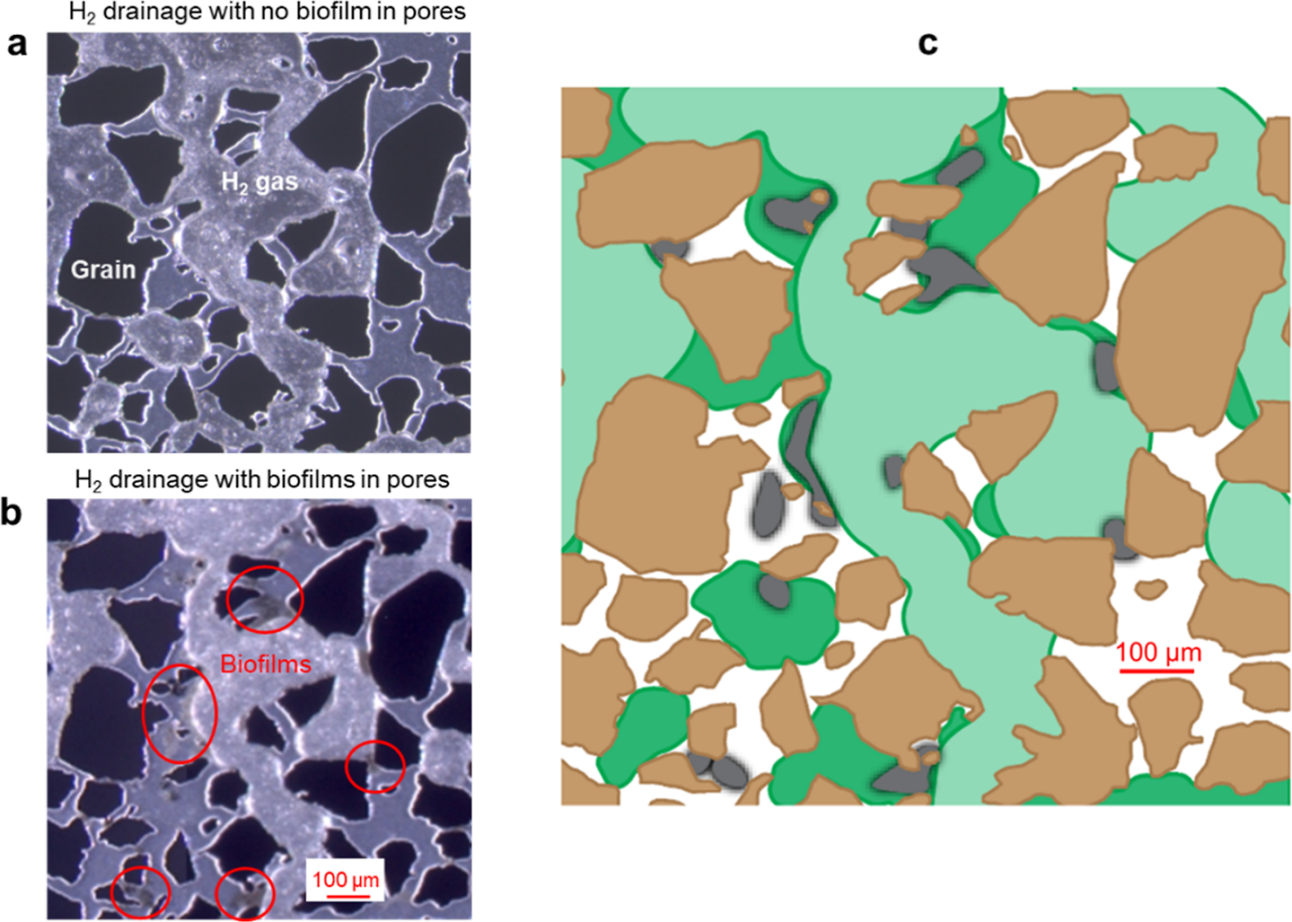
Effect of biofilms on H_2_ gas flow in a pore network initially filled with an aqueous bacterial solution. (a), The microscope image near the inlet showing the bacterial solution with H_2_ gas after 3 days. (b) The microscope image from the same location showing bacterial cell growth with lactate solution for 3 days, followed by H_2_ gas injection. (c), The conceptual image illustrates the H_2_ gas distribution with biofilms present was different from H_2_ gas distribution without biofilms. Dark green represents H_2_ gas in image (a), and light green in image (b). Brown indicates silicon grains from the microfluidic chip, white is the bacterial solution, and gray represents biofilms. Bioclogging predominantly in pore throats obstructed the H_2_ gas flow and displacement of the aqueous phase.

Dense and dark biofilms formed in the pore network using lactate as electron donor (Figure S5), in line with observations from μ-dish experiments (cf. Figure 1). Bioclogging in terms of grain-attached biofilms was observed globally in the pore network when lactate was supplied as the electron donor under continuous injection conditions. Most biofilms were prevalent near the nutrient-rich inlet, leading to a reduction of H_2_ saturation by 19%. Local H_2_ distribution was also influenced by biofilms near the pore throats (Figure 4). Using H_2_ as the electron donor resulted in fewer and less dense biofilms and, in contrast to lactate, the single cells did not attach to the grain surfaces and remained motile. This difference between the studied electron donors in porous media qualitatively agreed with observations from the μ-dish experiment detailed above (cf. Section 3.1). The lactate solution was subjected to continuous flow, while H_2_ was injected into the system with no subsequent flow, which may have influenced biofilm formation differently in the two cases.

**Figure 5:**
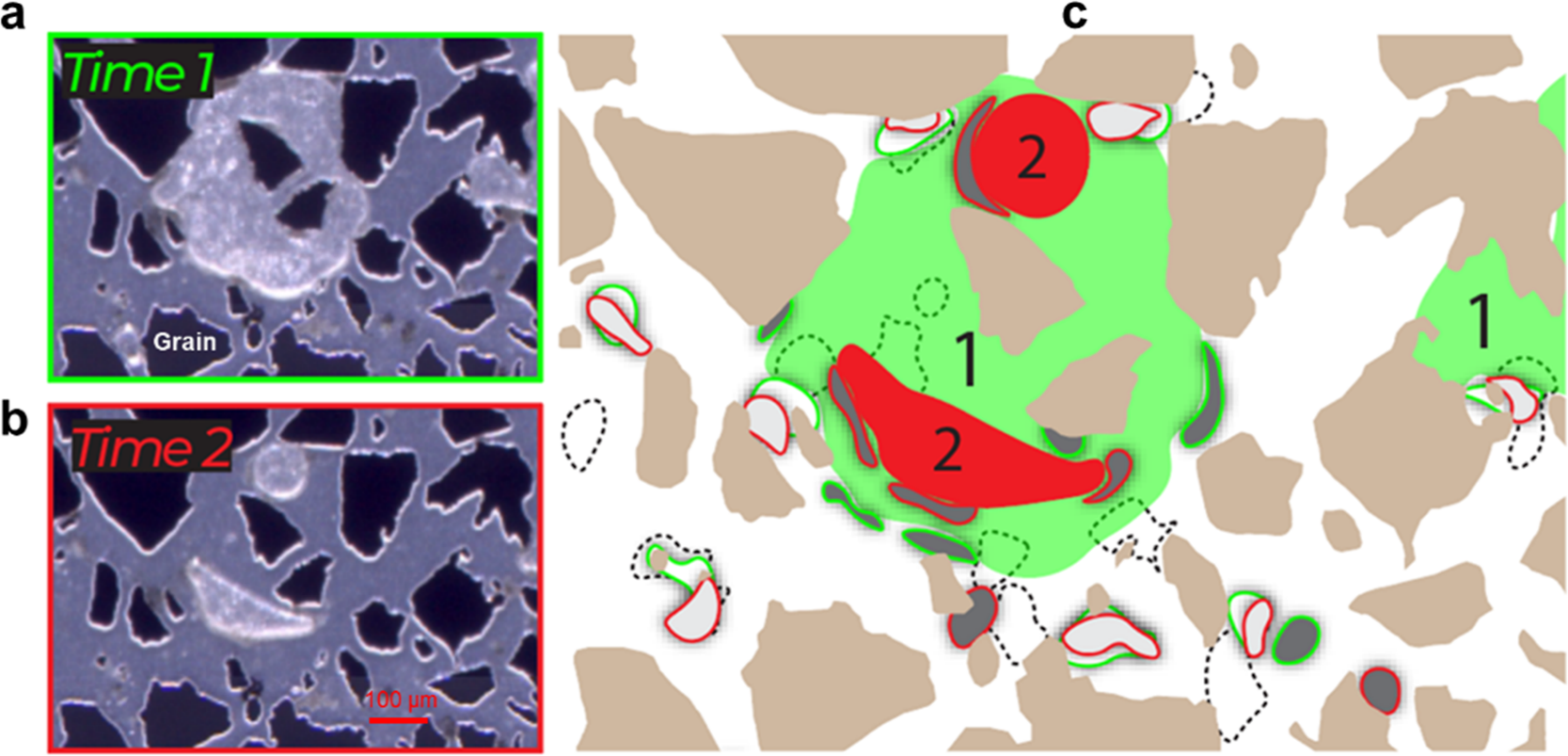
Pore-scale biofilm dynamics during H_2_ consumption in a pore network partially filled with H_2_ gas and an aqueous bacterial solution. (a) Microscope image of biofilms and H_2_ gas near the inlet at 6 h (time 1). (b) Microscope image at 36 h (time 2). (c) Conceptual image showing biofilm and H_2_ gas dynamics: Green represents H_2_ gas at time 1, red at time 2, brown represents silicon grains, and white is the bacterial solution. The temporal biofilm development is depicted at three times: immediately after H_2_ injection—dashed lines; time “1”—green outline; and time “2”—red outline. Two biofilm categories were identified in terms of their motility: surface-attached (white) and interface-chasing biofilms (gray). For the latter category, the biofilms followed the gas–liquid interface as the trapped H_2_ ganglia decreased in size between time 1 and 2 by microbial consumption. For the former category, the biofilms remained in place between times 1 and 2, largely adjacent to starting location (dashed lines). Note that the described biofilm dynamics was observed under no-flow conditions. Time-series pore images are provided in Figure S6.

During the shut-in period after biofilm formation in the pore network (cf. Section 2.3 for methodology), motile microbial cells aggregated first near the gas–liquid interfaces. In addition, the interface-chasing biofilms were linked to microbial interactions with trapped H_2_ ganglia (gray in Figure 5). The H_2_ ganglia size decreased over time due to microbial consumption, causing the gas–liquid interface to shift in the pore structure, as confirmed by the calculation in the Supporting Information S1. In response, motile microbial cells migrated from the biofilm matrix toward the new location of the gas–liquid interface in search of available H_2_, leading to biofilm dispersal and detachment. When biofilms detached from the grain surface, they were displaced by the advancing gas–liquid interface because of flow dynamics and interfacial forces in the pore network. By influencing both the spatial distribution and attachment strength of biofilms, this displacement collectively reduced bioclogging effects.

#### 3.2.2 Biofilm Detachment during Multiple Gas Injections

**Figure 6:**
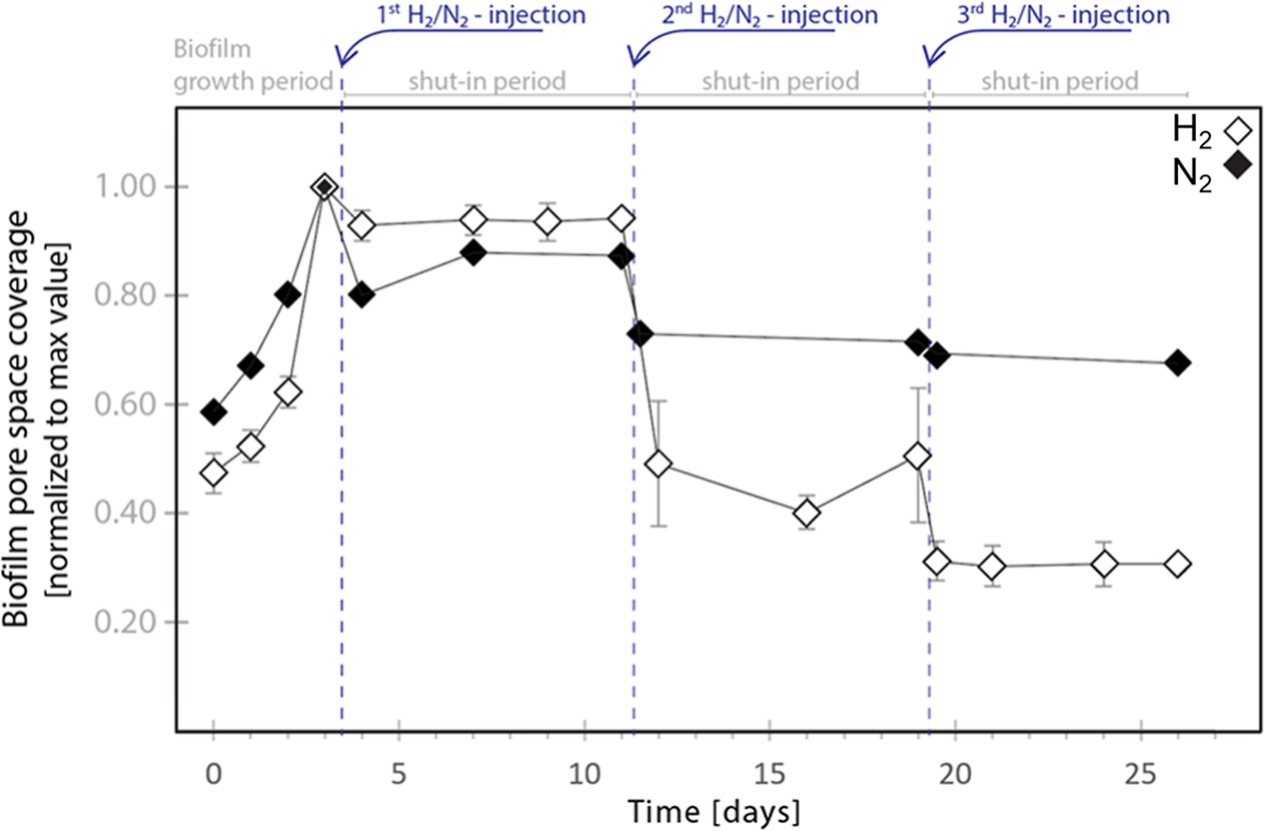
Effect of electron donors on induced biofilm detachment during a multistep gas drainage process. The growth period (0–3.5 days) with lactate increased the pore space biofilm coverage from its initial value. Biofilm coverage remained constant during the subsequent shut-in for H_2_, whereas a slight increase (0.87%) was observed for N_2_ at the end of the shut-in period, likely due to starvation promoting the biofilm formation. In the shut-in period after the second gas injection the biofilm coverage for H_2_ was on average 0.47, compared with 0.72 for N_2_. After the third gas injection, the biofilm coverage for H_2_ had decreased further (0.31), whereas the N_2_ case demonstrated a slight reduction only (0.68). Note that *y*-axis uses normalized biofilm pore space coverage calculated by dividing the biofilm coverage at each time point by the initial biofilm coverage, which was determined right after biofilm formation under lactate conditions for 3 days. The maximum coverage with N_2_ as the electron donor was 0.15 of the pore space, compared with 0.20 ± 0.12 for H_2_ (average of three repetitions ± one standard deviation).

The biofilm dynamics in porous media were quantified during multiple H_2_ injections (Figure 6) in the microfluidic pore network (cf. Section 2.3 for methodology). Baseline experiments were conducted using N_2_ injections following the same experimental protocols. Biofilm coverage reduced for both N_2_ and H_2_ after the first gas injection. N_2_ caused more detachment (20%) compared with H_2_ (11%) because of higher flow shear force (cf. Supporting Information S2). Microbial H_2_ consumption prompted biofilm fragments and cells to move with the gas bubble interface (cf. Figure 5), weakening the attachment of biofilms to the surface. Global analysis (i.e., analyzing all pores in the pore network) showed the following accumulated biofilm reduction (i.e., % of initial surface biofilms) during three subsequent H_2_ injections: 11% detached after the first injection, 55% detached after the second injection (44 percentage points decrease), and 69% detached after the third H_2_ injection (14 percentage points decrease). In comparison, the control experiments with N_2_ reduced biofilm coverage by 31% of the initial surface biofilms after three cycles, with higher flow shear forces although using the same volumetric injection rate. These results further support our interpretation that the biofilm detachment observed under H_2_ conditions is primarily driven by microbial interactions with H_2_ gas, rather than solely by physical gas/fluid flow effects. We note that a slight increase in biofilm coverage was observed after the first N_2_ injection, supporting prior observations that nutrient starvation can stimulate biofilm growth.

The reduced biofilm coverage during multicycle H_2_ gas injections supports the findings from the μ-dish and μ-channel experiments, while also introducing porous-media-specific effects: (i) external forces, such as flow shear forces, was the primary detachment mechanism during the first H_2_ injection; (ii) the increase in pH due to microbial H_2_ consumption and the detachment of biofilm fragments as they moved along the dynamic gas–liquid interfaces, weakened the biofilm attachment to the solid surface (Figure 5), which was the main mechanism during the second H_2_ injection; (iii) multiple gas injections increased displacement of weakly attached biofilms in the pore network. These compounded porous-media-specific effects come in addition to the dispersion of microbial cells by preferred planktonic lifestyle over biofilm formation when seeking access to H_2_ (Figure 3). The effect of higher shear forces on biofilm detachment diminished over cycles, with N_2_ injection causing minimal detachment in the second and third cycles. From this we can deduce that the larger biofilm reduction observed during the second injection of H_2_ was primarily from interactions between microbial cells and H_2_ gas. The amount of biofilm detachment decreased between the second and third cycles of H_2_ injection, from 44% in the second cycle to 14% in the third of H_2_ injection. This reduction was due to diminished microbial activity caused by the pH increase and the entrapment of residual biofilms in low-velocity regions (cf. the velocity field in Figure S3), which increased resistance to gas flow.

### 3.3 Implications for Underground H_2_ Storage

H_2_ is a versatile energy carrier and interesting for many industrial applications but is also a very common electron doner for subsurface microorganisms. It is expected that microbial communities will use the injected H_2_ during UHS as electron donor for diverse metabolisms, and induce a variety of microbial-triggered risks: (a) loss of the stored H_2_ and changes in gas composition,^22^ (b) risks to operational safety and deterioration in quality by H_2_S formation,^5^ (c) damage of the technical equipment by biocorrosion,^5^ (d) changes in near-well and reservoir properties by biofilm formation and precipitates.^9^ These adverse effects are influenced by the original composition of the in situ microbial community, the environmental diversity given by the geology and geochemistry of the reservoir, and the metabolic potential of each community. Hence, large-scale UHS deployment in porous reservoirs requires comprehensive and fundamental understanding of how and to what extent microbial processes affect storage sites, and the interplay between microbial, geochemical, and flow dynamics remains largely elusive.

While our study focuses on the mechanistic aspects using a single model bacterial strain, *O. alaskensis*, rather than the complex microbial communities found in actual storage sites, it provides critical insights into the fundamental processes at play. The controlled experimental conditions allow us to isolate and understand specific interactions between the SRB and the storage environment. This approach, although simplified, enables us to identify key factors that influence microbial behavior, such as biofilm formation and attachment/detachment dynamics.

Our study demonstrates that the choice of electron donor—H_2_ or lactate—affects microbial behavior and biofilm formation during UHS. Specifically, H_2_ promotes planktonic growth, while lactate enhances biofilm formation. When H_2_ gas is the sole electron donor, cells exhibit increased motility, allowing the microbe to disperse actively within a UHS storage site upon sensing H_2_. This migration toward the low soluble H_2_ gas results in increased consumption, but the increased motility reduces biofilm formation to lower the risk of bioclogging. The limited biofilm formation observed during our H_2_ experiments suggests that the risk of permeability reduction caused by biofilm formation by this SRB strain is low. Other H_2_-consuming microbes should be investigated to see if the increased mobility is indeed a general effect.

With relevance to the cyclic UHS site operation, our results demonstrate that multiple cycles of H_2_ drainage induce significant biofilm detachment, suggesting a reduced risk of microbial-induced bioclogging and decreased H_2_ injectivity. However, the cyclic injection process tends to push microbial cells and biofilm fragments into low-velocity regions within the reservoir, where the pore structure protects the biofilm by reducing flow shear forces. This redistribution can complicate efforts to mitigate microbial activity, as these regions often provide favorable conditions for microbial survival, including access to residual H_2_ and nutrients. Residual gas not only represents a loss in storage efficiency but also serves as a continuous source of nutrients for the residual microbial population. Additionally, the reservoir rocks can buffer pH increases during microbial consumption, potentially enabling the long-term establishment of a persistent microbial population capable of reforming biofilms.

Our findings highlight the necessity for careful site-specific evaluations in real-world UHS projects. The observed microbial growth, gas consumption, and biofilm dynamics under controlled conditions point to potential risks and behaviors that could manifest in more complex, natural settings. By understanding these processes in a simplified model, we can better predict and mitigate microbial risks in actual storage sites. Moreover, our study underscores the importance of addressing knowledge gaps in microbial interactions within UHS environments. Future research should aim to incorporate diverse microbial communities, mixed-electron donors, and varying environmental conditions to build on our findings. This will enhance the predictive power and applicability of microbial risk assessments, ultimately contributing to safer and more efficient underground hydrogen storage solutions.

## Supporting Information Available

Shear forces of hydrogen and nitrogen gas flow in the pore network (S1); hydrogen diffusion loss and Ostwald ripening in the microfluidic pore network (S2); experimental scheme for bacterial incubation (Figure S1); microbial responses to H_2_ gas (Figure S2); pore network and experimental setup (Figure S3); image segmentation for microfluidic experiment analysis (Figure S4); differences in biofilm formation between H_2_ gas and lactate solution (Figure S5); differences in gas drainage-induced biofilm detachment (Figure S6) (PDF)
