# Supplementary material for: Growth on Hydrogen by the Sulfate-Reducing Oleidesulfovibrio alaskensis Induces Biofilm Dispersion and Detachment—Implications for Underground Hydrogen Storage
Source: Environ Sci Technol. 2025 Apr 4;59(14):7095–105. doi: 10.1021/acs.est.4c13893 (PMC12004927; doi:10.1021/acs.est.4c13893)
Supplement: Supplementary file 1 — es4c13893_si_001.pdf [file es4c13893_si_001.pdf]

Supporting Information for

Growth on hydrogen by the sulfate-reducing *Oleidesulfovibrio alaskensis* induces biofilm dispersion and detachment – implications for underground hydrogen storage

Na Liu[1], Christian Ostertag-Henning[2], Martin A. Fernø[1,3], Nicole Dopffel[3]*

1 Department of Physics and Technology, University of Bergen, Allegaten 55, 5007, Bergen, Norway

2 Bundesanstalt für Geowissenschaften und Rohstoffe, Geozentrum Hannover Stilleweg 2, 30655, Hannover, Germany

3 Norwegian Research Centre AS – NORCE, Nygårdsgaten 112, 5008, Bergen, Norway

*Corresponding author: Na LIU, Email: Na.Liu@uib.no

Supporting information contains 10 pages with 6 figures

**Contents**	**Pages**
**S1:** Hydrogen diffusion loss and Ostwald ripening in the microfluidic pore network	S2
**S2:** Shear forces of the hydrogen gas and nitrogen gas flow in the pore network	S3
**Figure S1.** The experimental scheme involved incubating approximately 1.5 mL of precultured bacterial solution (BS) in a μ-Dish.	S4
**Figure S2.** Microbial responses to H_2 gas.	S5
**Figure S3.** Pore network and experimental setup.	S6
**Figure S4.** Image segmentation for microfluidic experiment analysis**.**	S7
**Figure S5**. Differences in biofilm formation between H_2 gas (top row) and lactate solution (bottom row) in the pore network.	S8
**Figure S6**. Differences in gas drainage induced biofilm detachment.	S9
**References**	S10

S1: Hydrogen diffusion loss and Ostwald ripening in the microfluidic pore network

To distinguish between hydrogen dissolution and microbial consumption, we calculated the diffusion time (t) for hydrogen throughout the micromodel under experimental conditions, based on Fick's laws of gas diffusion [1]:

$$t \approx \frac{\pi}{D_e\varphi}(\frac{l}{2})^2 \;=\; 31.25\ \text{min}$$

where φ is the porosity of the pore network, D_e is the effective diffusion coefficient (≈ 4.29 ×10^{-9} m^2/s in a random homogeneous porous media saturated with pure water at 25 °C) [2-4], and the diffusion distance *l* equals the longest distance between 100% H_2 phase and 100% aqueous phase in the pore network (measured to be approximately 2.5 mm in our micromodel). In our experiments, following breakthrough, an additional 100 pore volumes of H_2 gas were injected over four hours. This ensured the dissolved H_2 concentration in the aqueous phase stabilized at the experimental temperature and pressure.

Ostwald ripening typically causes larger bubbles to grow while smaller ones shrink due to surface energy differences [5]. Detailed observations of bubble dynamics made in our work showed, however, a uniform decrease in both large and small bubbles over time. This indicates that Ostwald ripening was not a dominant effect. Therefore, we conclude that the primary mechanism for H_2 loss is microbial consumption rather than dissolution or Ostwald ripening.

S2: Shear forces of the hydrogen gas and nitrogen gas flow in the pore network:

Shear forces generated by nitrogen and hydrogen gas flow are relatively low compared to those of water under our experimental conditions. The shear stress (τ) can be estimated using the following equation [6]:

$$\tau = \mu \frac{4Q}{\pi R^3}$$

where τ is shear stress (Pa), Q is volumetric flow rate (m^3/s), μ is dynamic viscosity of the fluid (Pa·s), R is hydraulic radius of the pore (m). The volumetric flow rate (Q) was set to 5 µL/min for both gas injections. Under the experimental conditions, the following viscosities were used: Hydrogen gas: 89 µPa·s; Nitrogen gas: 186 µPa·s. The shear stress for hydrogen gas flow was calculated to be approximately 279.69 Pa, while for nitrogen gas flow, it was 584.51 Pa—nearly twice as high as the shear stress for hydrogen.

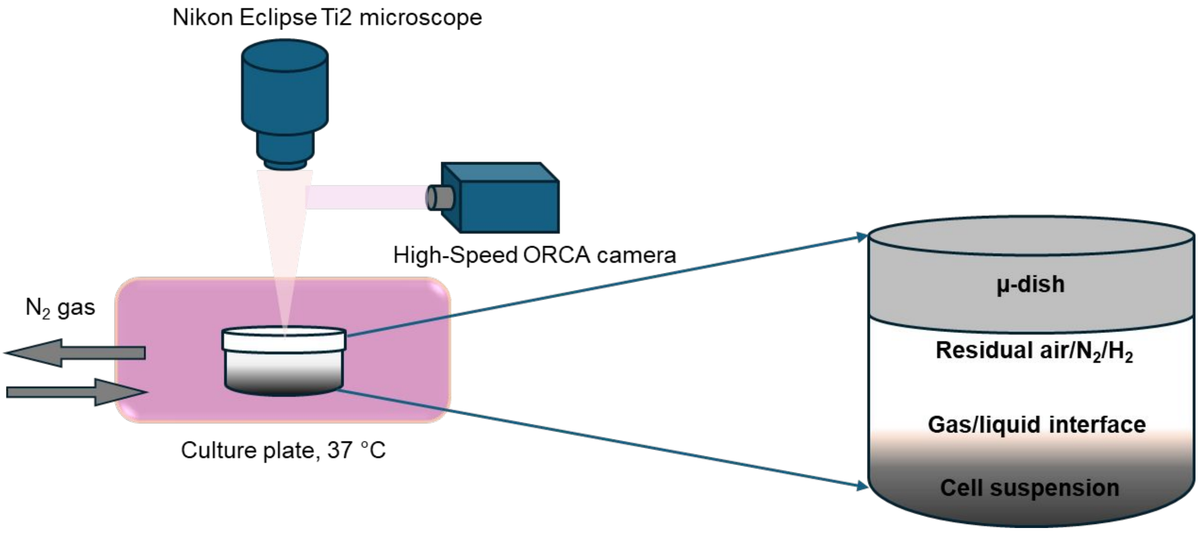

Figure S1. The experimental scheme involved incubating approximately 1.5 mL of precultured bacterial solution (BS) in a µ-Dish. For the no electron donor experiment, the headspace contained residual air, which is toxic to microbial growth. In the lactate experiment, the headspace was filled with N_2 gas, and the inoculated solution was a 50:50 mixture of BS and growth medium. In the H_2 experiment, the headspace was filled with pure H_2 gas, and the incubation solution consisted of 100% BS. The solution depth was maintained at approximately 25.2 µm across all experiments.

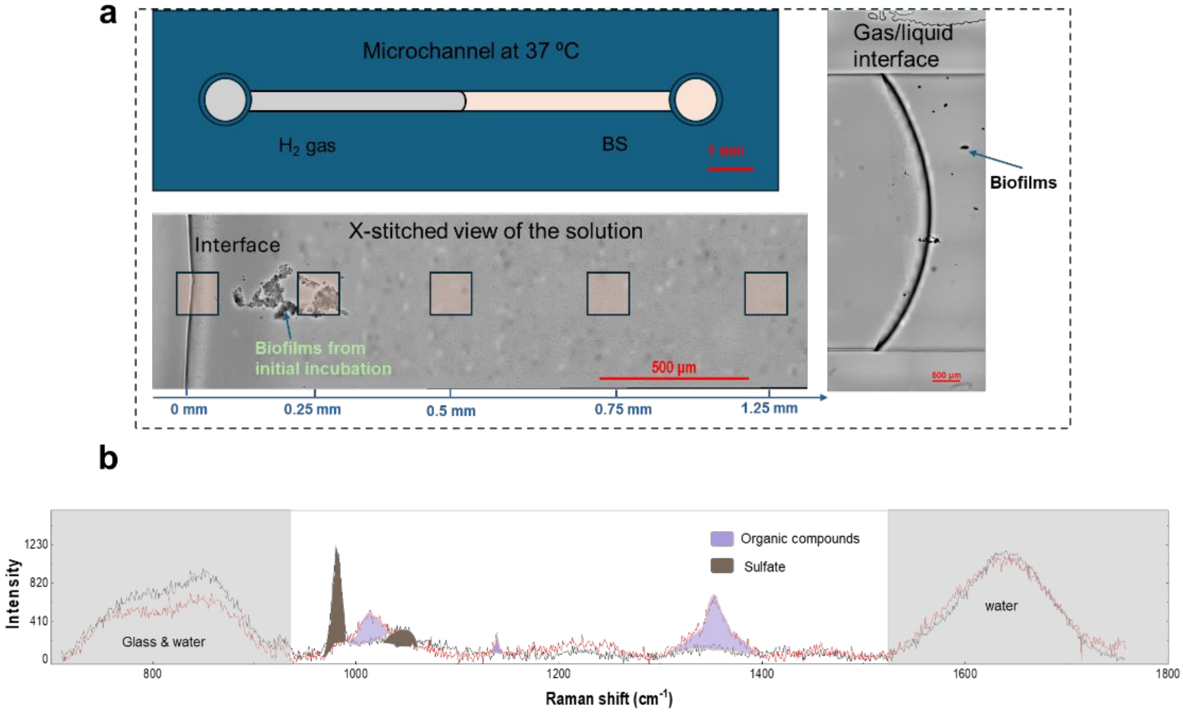

Figure S2. Microbial responses to H_2 gas. (a) Left-top: Schematic representation of the experimental setup. One end of the microchannel was filled with H_2 gas and the other end with a dense bacterial solution, creating a gas-liquid interface in the middle of the microchannel. The microchannel was positioned within a controlled heating chamber mounted on a microscope, enabling *in situ* observation of microbial cell motility over a period of 4 days. The microchannel was initially cultured in a heating cabinet at 37°C for approximately 16 hours before being transferred to a microscope for in situ observation of cell mobility and changes at the interface. Left-bottom: Image of the x-stitched view in the solution from the interface. The squares in the image indicate the positions where videos were recorded to capture microbial cell movement, enabling analysis of cellular motility in relation to proximity to the gas-liquid interface. The right image is the gas-liquid interface within the microchannel. (b) Sulfate consumption during the microbial sulfate-reducing process. The 21.1 mM sulfate in the growth medium (red line) was depleted (brown regions) after culturing microbial cells (purple regions) in the presence of an 80:20 H_2/CO_2 gas mixture (black line).

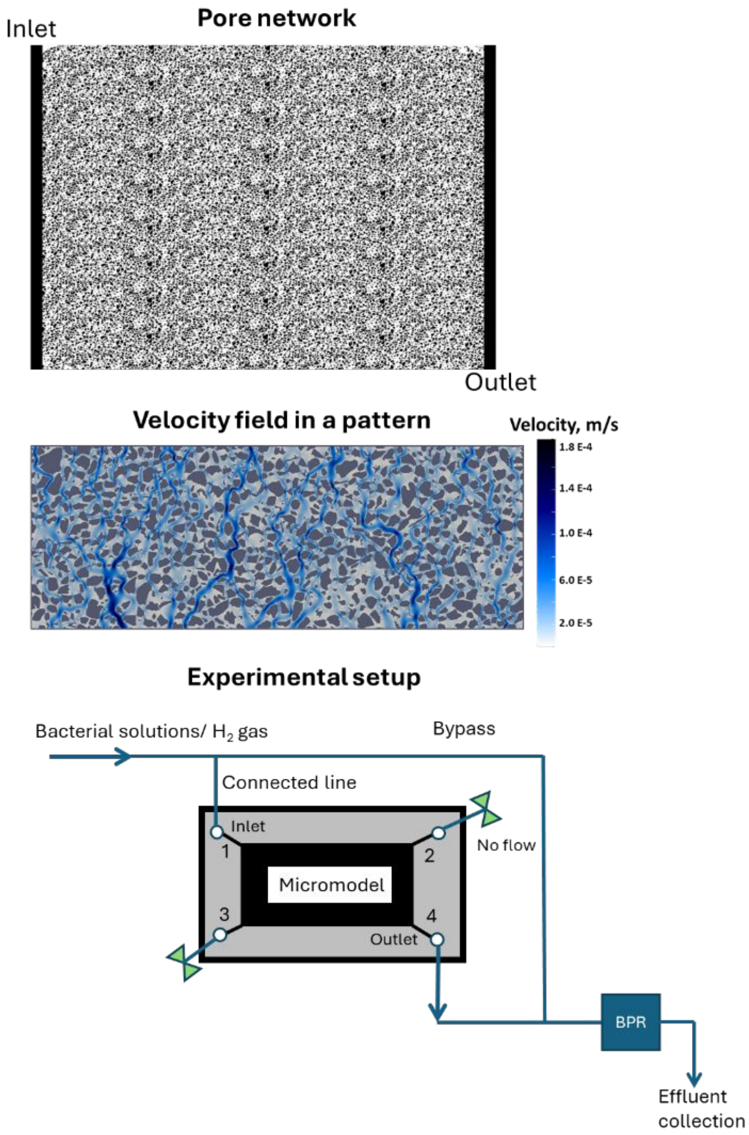

Figure S3. Pore network and experimental setup. Top row: The pore network of micromodel was constructed based on a thin section obtained from an authentic sandstone rock sample. The porous pattern constitutes 36 repetitions (arranged in a 4 × 9 configuration) of a smaller unique pattern. Middle row: A simulation was performed to visualize the magnitude of steady-state flow velocity within the small unique pattern (confer to https://daavid00.github.io/pymm/examples.html#image for details on the simulation setup). Bottom row: A simplified schematic of the experimental setup illustrates the intended flow into the pore network.

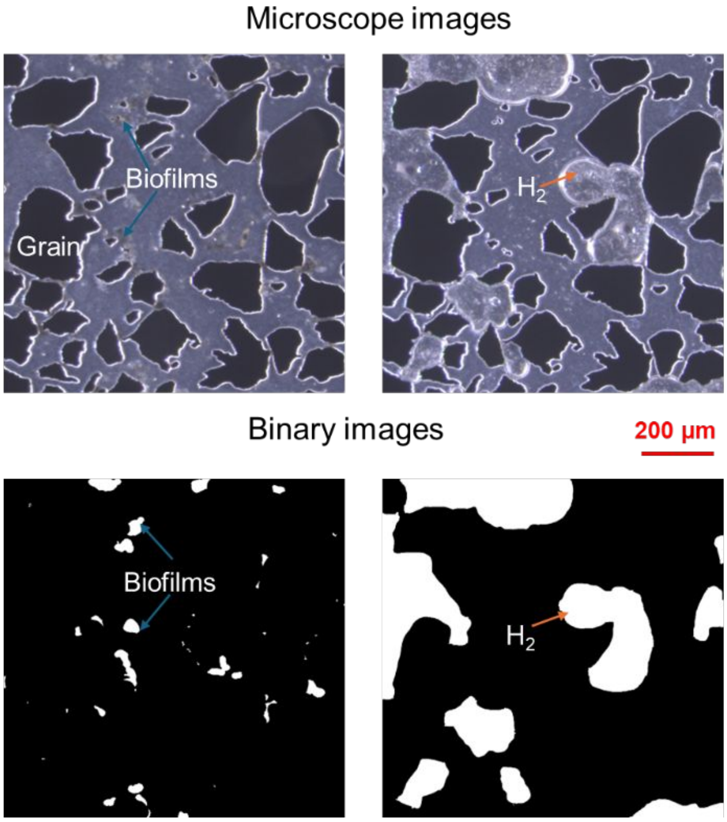

Figure S4. Image segmentation for microfluidic experiment analysis. Top row: Original microscope images showing biofilms and H_2 gas bubbles within the pores. Bottom row: Segmented binary images highlighting only the biofilms and H_2 gas bubbles. Image segmentation and subsequent analysis were performed using a custom Python algorithm built with *OpenCV* [7]. The complex pore network was divided into 288 individual units using *Patchify*, with each unit exhibiting a porosity range of 0.59 to 0.66. For each unit, background images—captured at the start of the experiment without biofilms or gas—were subtracted from the acquired images, followed by grayscale conversion. Biofilm segmentation was achieved through thresholding, which distinguished dark and bright pixels. For gas-phase segmentation, after background removal, the laminar interface was extracted to isolate the gas phase from its surroundings. Pore space was segmented removing the dark silicon grains from the image. Quantitative evaluation of the pore network's porosity involved summing the pore space pixels within each unit and dividing by the total image size. Biofilm coverage was determined by computing the ratio of biofilm pixels to the total pore space. Similarly, gas saturation was calculated as the ratio of gas-phase pixels to the total pore space.

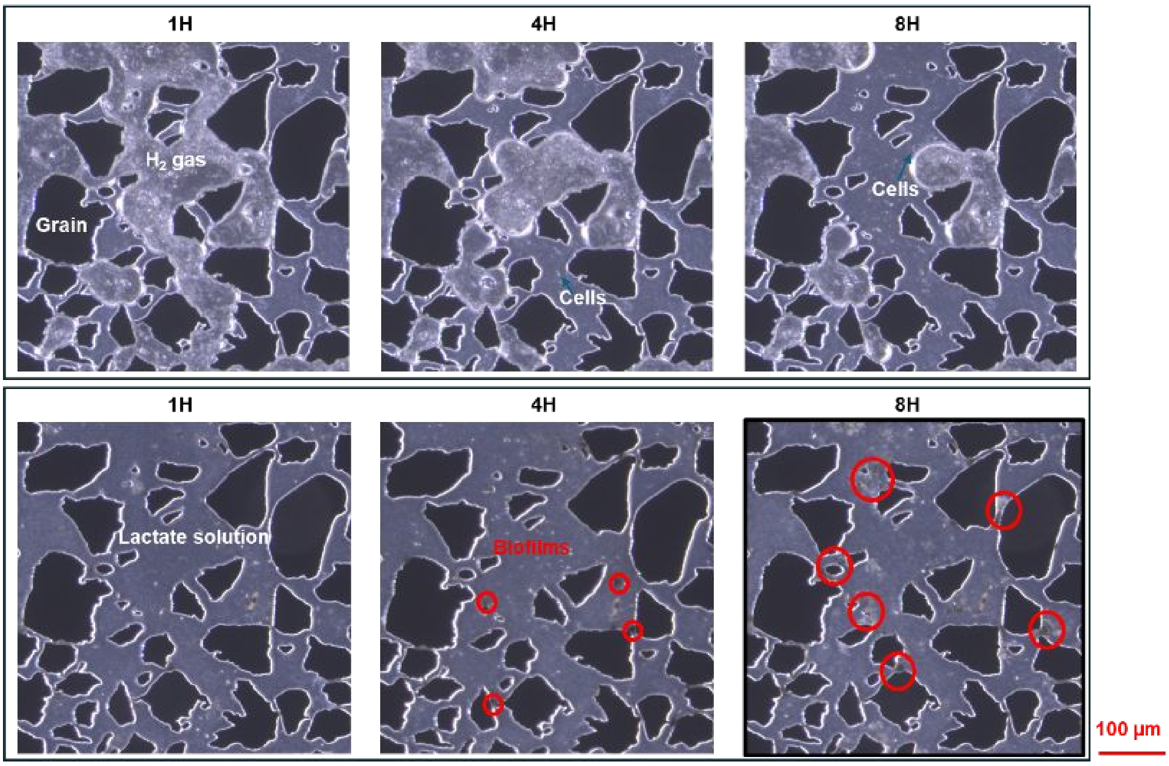

Figure S5. Differences in biofilm formation between H_2 gas (top row) and lactate solution (bottom row) in the pore network. In the microscope images, biofilms appear as dense, structured clusters of white or dark pixels attached to the silicon surface or gas/liquid interfaces, indicated by red circles, while planktonic cells are seen as isolated white pixels. When H_2 gas served as the electron donor, it was gradually consumed by microbial cells; however, this resulted in little biomasses production. In contrast, when approximately 10 mM lactate was used as the growth substrate, biofilm formation was evident over time, with most biofilms accumulating in the pore throats and leading to significant clogging.

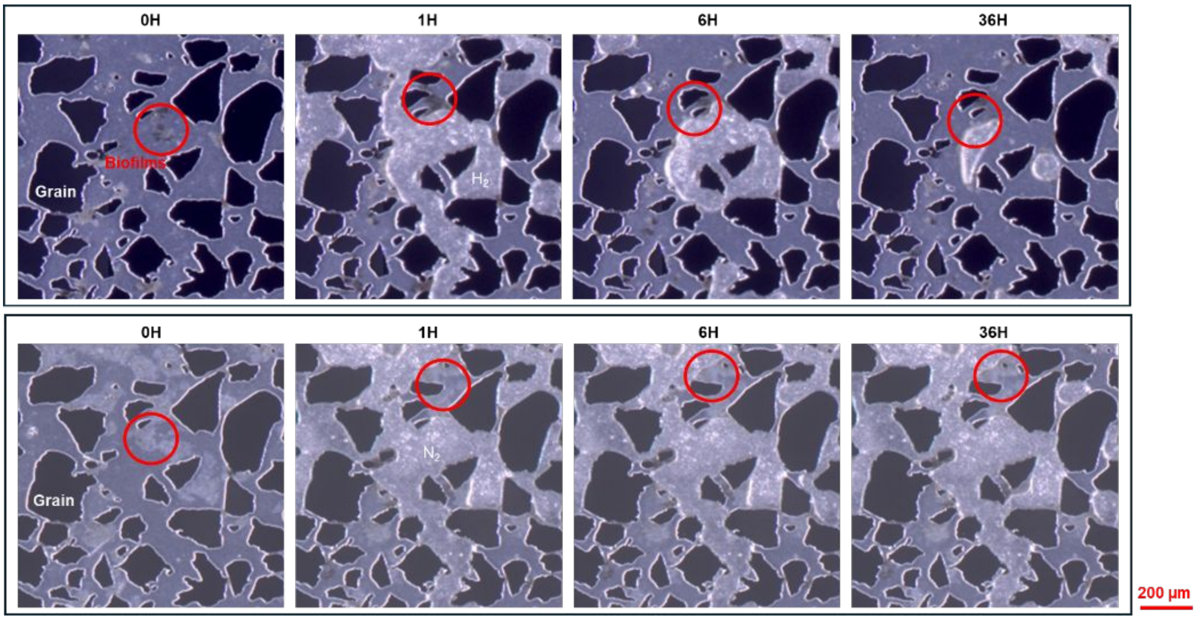

Figure S6. Differences in gas drainage induced biofilm detachment. Top: Time-lapse series of biofilm detachment with H_2 gas: Initially, biofilms in the pore throats obstructed the entry of H_2 gas into the pore space. As the H_2 gas bubbles shrank due to microbial consumption, segments of the biofilm detached from the pore throats and migrated with the H_2 gas to access nutrients. This migration weakened their attachment to the solid surfaces. During a subsequent gas invasion, they were dislodged from the pore network. Bottom: Time-lapse series of biofilm development with N_2 gas: Unlike with H_2, microbial cells did not consume the trapped N_2 gas or accumulate at the N_2 gas interface. Consequently, the injection of N_2 gas caused minimal biofilm detachment over time. However, biofilm detachment can still occur due to shear forces by the gas flow.

Reference:

1. Crank, J., *The mathematics of diffusion*. Oxford university press: 1979.
2. Hemme, C.; Van Berk, W., Hydrogeochemical Modeling to Identify Potential Risks of Underground Hydrogen Storage in Depleted Gas Fields. *Applied Sciences* **2018,** *8*, (11), 2282.
3. Shen, L.; Chen, Z., Critical review of the impact of tortuosity on diffusion. *Chemical Engineering Science* **2007,** *62*, (14), 3748-3755.
4. Neale, G. H.; Nader, W. K., Prediction of transport processes within porous media: diffusive flow processes within an homogeneous swarm of spherical particles. *AIChE Journal* **1973,** *19*, (1), 112-119.
5. Voorhees, P. W., The theory of Ostwald ripening. *Journal of Statistical Physics* **1985,** *38*, (1), 231-252.
6. Kirby, B. J., *Micro- and Nanoscale Fluid Mechanics: Transport in Microfluidic Devices*. Cambridge University Press: 2010.
7. Benali, B.; Sæle, A.; Liu, N.; Fernø, M. A.; Alcorn, Z. P., Pore-level Ostwald ripening of CO2 foams at reservoir pressure. *Transport in Porous Media* **2023**.
